# Pregnancy-Specific Glycoprotein 9 Enhances Store-Operated Calcium Entry and Nitric Oxide Release in Human Umbilical Vein Endothelial Cells

**DOI:** 10.3390/diagnostics13061134

**Published:** 2023-03-16

**Authors:** Ying Qin, Qinggang Meng, Qunhua Wang, Mingzhu Wu, Yan Fang, Chengcheng Tu, Xinyang Hu, Bing Shen, Hongbo Chen, Xiaohong Xu

**Affiliations:** 1 Clinical Laboratory, Anhui No. 2 Provincial People’s Hospital, Hefei 230011, China; qinying19881012@163.com; 2 School of Basic Medicine, Anhui Medical University, Hefei 230032, China; 13905694425@163.com (X.H.); shenbing@ahmu.edu.cn (B.S.); 3 Department of Obstetrics and Gynecology, Maternal and Child Health Hospital, The Affiliated Hospital of Anhui Medical University, Hefei 230032, China; mengqinggang@stu.ahmu.edu.cn (Q.M.); wumingzhu@stu.ahmu.edu.cn (M.W.); 2145012060@stu.ahmu.edu.cn (Y.F.); ahmu_tu@163.com (C.T.); 4 Department of Obstetrics and Gynecology, The Fist Affiliated Hospital of USTC, Anhui Provincial Hospital, Hefei 230032, China; wqh971234@163.com; 5 Department of Clinical Laboratory, Maternal and Child Health Hospital, The Affiliated Hospital of Anhui Medical University, Hefei 230032, China

**Keywords:** preeclampsia, pregnancy-specific glycoprotein 9, endothelial function, nitric oxide synthase, calcium store manipulates calcium influx

## Abstract

We explored changes in pregnancy-specific glycoprotein 9 (PSG9) levels in the serum of patients with preeclampsia and the effects and underlying mechanisms of PSG9 effects on calcium (Ca^2+^) homeostasis and nitric oxide (NO) release in human umbilical vein endothelial cells (HUVECs). Western blotting was used to detect protein expression levels, and an NO fluorescence probe was used to examine NO production. Intracellular Ca^2+^ concentrations were measured using a Ca^2+^-sensitive fluorescent dye under a fluorescence microscope. Compared with those in healthy pregnant women, serum PSG9 levels were significantly decreased in patients with preeclampsia. PSG9 (0.1 μg/mL) treatment of HUVECs significantly enhanced the expression levels of store-operated calcium entry (SOCE) channel proteins Orai1 and Orai2, but not Orai3, and of endothelial nitric oxide synthase (eNOS) and NO production. Pretreatment with an inhibitor of SOCE (BTP2) abolished PSG9-enhanced Orai1, Orai2, and eNOS expression levels and NO production in HUVECs. The mechanisms underlying SOCE that were PSG9 enhanced in HUVECs appear to involve the Ca^2+^/eNOS/NO signaling pathway. These findings suggest that serum PSG9 levels may be a potential biomarker for monitoring the occurrence or development of preeclampsia in pregnancy and that PSG9 may be a potential therapeutic target for the treatment of preeclampsia.

## 1. Introduction

Preeclampsia is a hypertensive disorder complicating pregnancy. It presents as hypertension after 20 weeks of pregnancy and is accompanied by abnormal urine protein and damage to the kidney, liver, or other organs [1,2]. The current global incidence of preeclampsia is 2–8% [3]. Adverse fetal pregnancy outcomes, such as fetal growth restriction and premature delivery, are closely related to preeclampsia, which is one of the main causes of maternal mortality [4]. The primary factors leading to preeclampsia are the insufficient remodeling of uterine spiral arteries, over-activation of the inflammatory immune response, damaged vascular endothelial cells, genetic factors, and nutritional deficiency [5]. Although the pathogenic factors leading to preeclampsia are still unclear, it is well known that systemic vasospasm and vascular endothelial injury are the basic pathophysiological changes [6,7,8,9,10]. Pregnancy-specific glycoproteins (PSGs) are an immunoglobulin superfamily that includes 10 subtypes (PSG1–9, PSG11) and are secreted mainly from trophoblasts in maternal blood during human pregnancy [11]. Most of the reports on PSG9 emphasize its role in tumorigenesis [12,13,14] although a recent study indicated that PSG9 promotes angiogenesis by endothelial cells [15]. However, an association between PSG9 and the occurrence or development of preeclampsia remains unclear.

The vascular endothelium is a monolayer of epithelial cells covering the inner surface of blood vessels and plays a variety of roles in regulating blood vessel tension and blood coagulation. Nitric oxide (NO) is produced by the vascular endothelium and acts on the underlying vascular smooth muscle cells to reduce tension and relax blood vessels [16]. The inability of uterine artery endothelial cells to produce a large amount of NO is a sign of insufficient vasodilation in pregnant women with preeclampsia [17]. The production of NO directly depends on the expression level of endothelial nitric oxide synthase (eNOS) in endothelial cells and the concentration of Ca^2+^ in the cytoplasm [16]. Store-operated Ca^2+^ entry (SOCE) is a mechanism that mediates extracellular Ca^2+^ entry into cells, an important physiological function [18]. Orai channel proteins are the major components of SOCE and are responsible for the influx of Ca^2+^ across the cell membrane. There are three Orai subtypes: Orai1, Orai2, and Orai3 [19]. All three subtypes are activated by the depletion of Ca^2+^ stores, which is often induced by G protein-coupled receptor activation or following the release of Ca^2+^ stores by inositol triphosphate receptors located in the endoplasmic reticulum (ER). SOCE-mediated Ca^2+^ influx may induce NO production and release from endothelial cells [20,21,22]. However, it is unclear whether PSG9 regulates Orai channels and NO release from endothelial cells.

Because the placenta is directly linked to the maternal circulation, abnormalities in the expression of placental factors in women with preeclampsia can be detected in maternal serum protein levels. In a previous study by our team, we identified differentially expressed proteins in blood samples derived from patients with preeclampsia by using isobaric tags for relative and absolute quantitation (iTRAQ) combined with high-performance liquid chromatography–tandem mass spectrometry (LC-MS/MS) [23]. Compared with those in healthy pregnant women, PSG9 levels were significantly decreased in blood samples collected from pregnant women with preeclampsia. In the present study, we used Western blotting, an NO fluorescence probe, intracellular Ca^2+^ concentration ([Ca^2+^]_i_) measurement assays, and in vivo blood vessel tension measurement methods to investigate the effect of PSG9 on NO production in human umbilical vein endothelial cells (HUVECs) and on agonist-induced umbilical vein relaxation. 

## 2. Materials and Methods

### 2.1. Participants and Clinical Samples

All patients provided written informed consent for this study, which was approved by the Medical Research Ethics Committee of Anhui Medical University (Anhui Medical Ethics approval no. 20150192). Blood samples were collected from December 2018 to September 2020 at the Maternal and Child Health Hospital affiliated with Anhui Medical University. The criteria for the diagnosis of preeclampsia were consistent with the 2019 American College of Obstetricians and Gynecologists pregnancy hypertension guidelines [3]. All pregnant women were primiparas without infection or gestational diabetes.

### 2.2. Cell Culture

HUVECs were purchased from Shanghai Guandao Bioengineering Co., Ltd. (item number: C396, Shanghai, China). HUVECs were cultured in Roswell Park Memorial Institute (RPMI)-1640 medium supplemented with 10% fetal bovine serum (Gibco, Thermo Fisher Scientific, Waltham, MA, USA) at 37 °C in a 5% CO_2_ incubator.

### 2.3. Western Blotting

The cultured cells were lysed on ice with RIPA buffer (50 mM Tris, pH 7.4; 150 mM NaCl, 1% Triton X-100, 1% sodium deoxycholate, 0.1% SDS and sodium orthovanadate, sodium fluoride, EDTA, leupeptin, and other protease inhibitors) and then centrifuged at 12,000 rpm and 4 °C for 20 min. The resultant proteins underwent electrophoresis using sodium dodecyl sulfate–polyacrylamide gel electrophoresis and were transferred to polyvinylidene fluoride membranes. Then polyvinylidene fluoride membrane was incubated with the respective primary antibodies, including rabbit anti-PSG9 antibody (1:1000 dilution; GeneTex, Irvine, CA, USA), rabbit anti-Orai1 antibody, rabbit anti-Orai2 antibody, rabbit anti-Orai3 antibody (1:1000 dilution; Affinity Biosciences, Liyang, China), and rabbit anti-eNOS (1:1000 dilution; Cell Signaling Technology, Danvers, MA, USA), overnight at 4 °C. Target proteins were detected using a luminescent agent from an enhanced chemiluminescence system after incubation with horseradish peroxidase-labeled secondary antibody (1:5000 dilution, Elabscience Biotechnology, Wuhan, China). ImageJ software (National Institutes of Health, version 1.53, Bethesda, MA, USA) was used to analyze the optical density of the resulting bands. The values were normalized to the densitometric values of GAPDH (1:1000 dilution; Affinity Biosciences, China).

### 2.4. NO Fluorescence Probe

An NO fluorescent probe (DAF-FM DA, Beyotime, Shanghai, China) was used to evaluate the generation of intracellular NO. DAF-FM has only weak fluorescence, but it produces a strong fluorescence signal after reacting with NO that can be detected at an excitation wavelength of 495 nm. After the cells were treated with various agents, the cells on a glass slide were processed according to the instructions of the DAF-FM DA kit and eventually photographed under a fluorescence microscope (Nikon T200, Tokyo, Japan). Cell nuclei were identified by application of 4′,6-diamidino-2-phenylindole. The mean fluorescence intensity of NO was measured using ImageJ software (version 1.53). The mean fluorescence density was calculated by determining the value of the fluorescence intensity of interest divided by the total imaging area.

### 2.5. [Ca^2+^]_i_ Measurement

Fluo-8/AM (Abcam, Shanghai, China) is a widely used fluorescent Ca^2+^ indicator. Before binding to Ca^2+^, Fluo-8 produces little florescence. However, after Fluo-8 combines with Ca^2+^, the fluorescence intensity increases at least 200 times. Fluo-8 is excited by light at a wavelength of 488 nm using an argon ion laser, and the emitted light is at a wavelength of 514 nm. We incubated HUVECs for 20 min at 37 °C with 2 μM Fluo-8/AM and 0.02% pluronic F-127 (Invitrogen, Eugene, OR, USA). After the cells were washed with phosphate-buffered saline (PBS) to remove extracellular Fluo-8 dye, the cellular Ca^2+^ store was depleted by the application of thapsigargin (TG, 2 μM) in a Ca^2+^-free solution (OPSS, 140 mM NaCl, 5 mM KCl, 2 mM CaCl_2_, 1 mM MgCl_2_, 10 mM glucose, and 5 mM HEPES, pH 7.3 to 7.4 adjusted with NaOH). The Ca^2+^ influx was initiated by applying 2 mM Ca^2+^ to the medium. The baseline fluorescence intensity before extracellular Ca^2+^ application was considered F0. The peak fluorescence intensity after extracellular Ca^2+^ application was considered F1. The change in Ca^2+^ concentration in the cells is presented as a fluorescence intensity ratio (F1/F0) before and after the addition of extracellular Ca^2+^.

### 2.6. Statistical Analysis

The data were analyzed using GraphPad Prism software (version 7, San Diego, CA, USA). Statistical comparisons were performed using the two-tailed Mann–Whitney test or two-way analysis of variance followed by the Games–Howell post hoc tests when more than two treatments were compared. Unless otherwise stated, the data are presented as means with standard errors (mean ± SE). Values of *p* < 0.05 were considered statistically significant differences. 

## 3. Results

### 3.1. Changes in Serum PSG9 Levels in Patients with Preeclampsia

**Figure 1:**
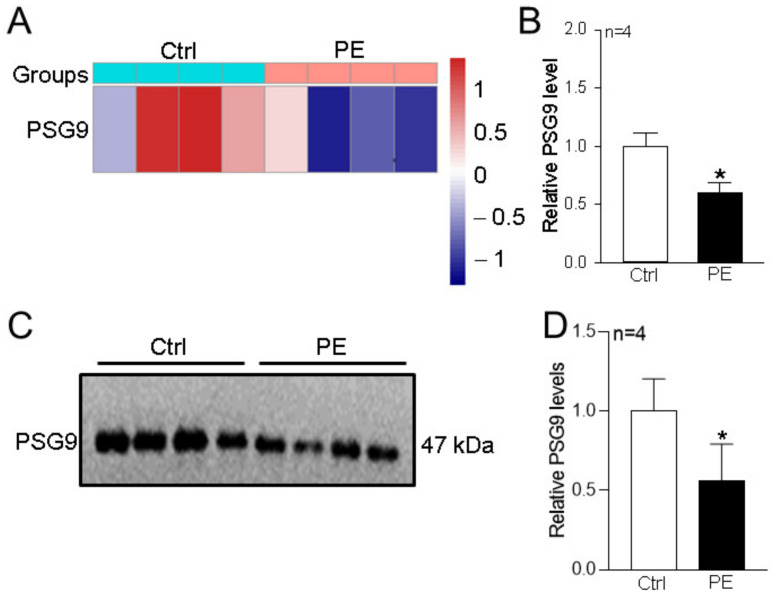
Changes in serum PSG9 levels in patients with preeclampsia. (**A**,**B**) Cluster thermographic analysis (**A**) and summary data (**B**) showing serum relative PSG9 expression levels identified by isobaric tags for relative and absolute quantitation combined with LC-MS/MS technology in healthy control (Ctrl) pregnant women and patients with preeclampsia (PE). Red indicates an increase in expression level, whereas blue indicates a decrease. The darker the color, the greater the change. (**C**,**D**) Representative Western blot image (**C**) and summary data (**D**) showing serum PSG9 expression levels in healthy control pregnant women and patients with preeclampsia. In (**B**) and (**D**), the mean PSG9 level for healthy control pregnant women served as the denominator, and all data were divided by this mean. Values are shown as the mean ± SEM (*n* = 4 per group). * *p* < 0.05 for PE vs. Ctrl.

In our previously published study, serum PSG9 levels identified by LC-MS/MS were significantly decreased in the patients with preeclampsia compared with those in the healthy control pregnant women (Figure 1A,B) [23]. To confirm this finding, in the present study, we collected additional samples from four patients with preeclampsia and from four healthy control pregnant women. The patients with preeclampsia had significantly higher systolic blood pressure and diastolic blood pressure than the healthy control women, but the ages and weeks of gestation did not significantly differ between the two groups (Table 1). 

Western blotting analyses were used to verify the PSG9 serum level changes in patients with preeclampsia. Our data showed that serum PSG9 protein levels were significantly decreased in the patients with preeclampsia compared with those in the healthy controls (Figure 1C,D). These results suggested that the secretion of PSG9 may be suppressed in patients with preeclampsia and may be related to the occurrence or development of preeclampsia.

### 3.2. Effects of PSG9 on the Expression Levels of Orais and on SOCE in HUVECs

**Figure 2:**
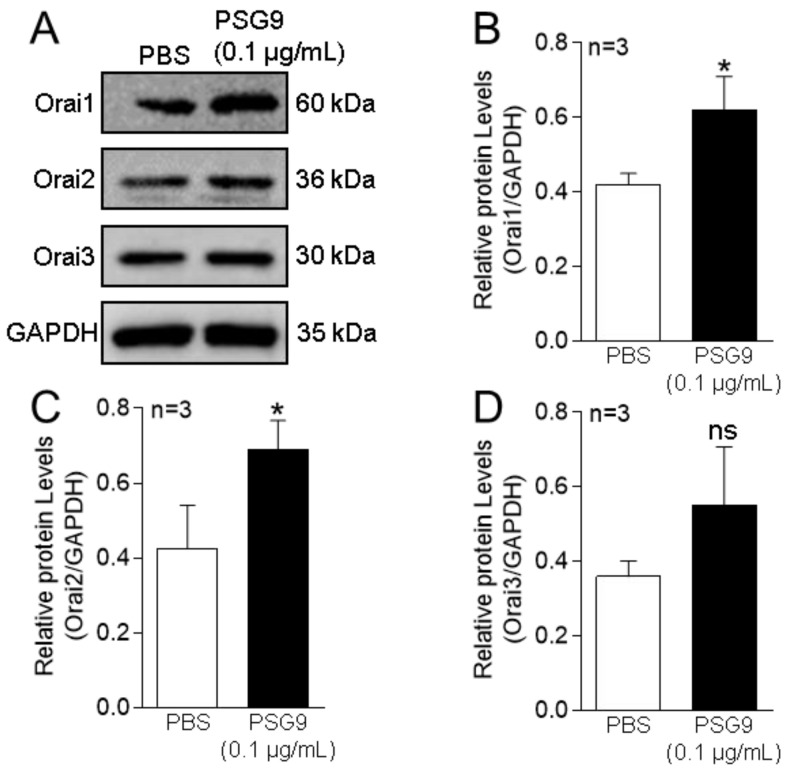
Effect of PSG9 on Orais expression in human umbilical vein endothelial cells (HUVECs). (**A**–**D**) Representative Western blot images (**A**) and summary data (**B**–**D**) showing Orai1, Orai2, and Orai3 expression levels after application of phosphate-buffered saline (PBS) or 0.1 μg/mL PSG9 for 24 h in HUVECs. Values are shown as the mean ± SEM (*n* = 3 as indicated). * *p* < 0.05 compared with PBS; ns, no significant difference.

**Figure 3:**
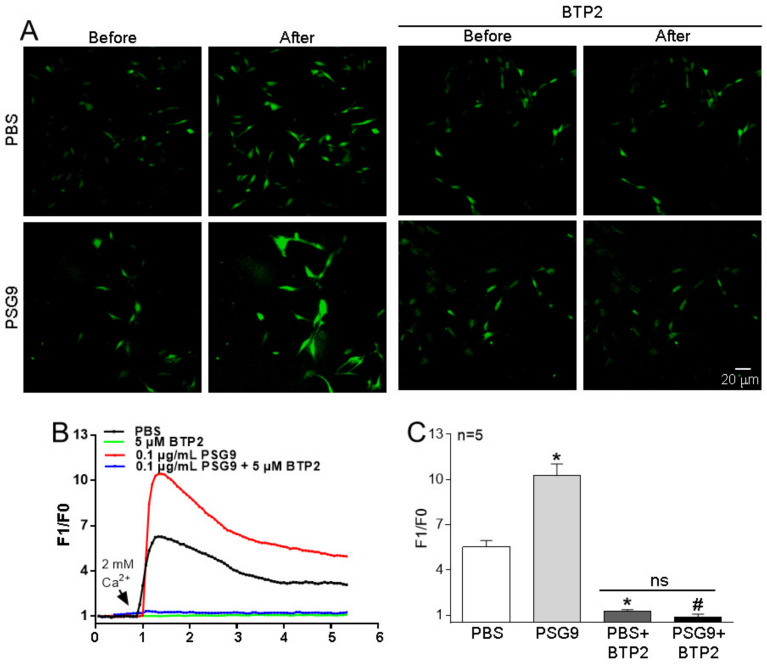
Effect of PSG9 on store-operated Ca^2+^ entry (SOCE) in human umbilical vein endothelial cells (HUVECs). (**A**–**C**) Representative images (**A**), traces (**B**), and summary data (**C**) showing SOCE-mediated intracellular Ca^2+^ rising after application of PBS, BTP2 (5 μM) + PBS, 0.1 μg/mL PSG9, and 0.1 μg/mL PSG9 + BTP2 (5 μM) in HUVECs. To assess SOCE, HUVECs were placed in a Ca^2+^-free solution, and the Ca^2+^ stores were depleted by application of 2 μM thapsigargin. SOCE was evoked by application of 2 mM Ca^2+^ to the medium. Values are shown as the mean ± SEM (*n* = 5 as indicated). * *p* < 0.05 compared with PBS; ^#^ *p* < 0.05 compared with PSG9; ns, no significant difference.

Many studies have shown that eNOS production and NO release are closely related to intracellular Ca^2+^ homeostasis [16,18]. SOCE controlled by the intracellular Ca^2+^ pool is an important pathway mediating extracellular Ca^2+^ influx and participates in a myriad of physiological and biochemical processes of various cells. The Orai family proteins make up the Ca^2+^ channel in the SOCE system. To investigate the effect of PSG9 on SOCE in HUVECs, we treated the cells with 0.1 μg/mL PSG9 for 24 h. Our Western blotting data showed that the application of PSG9 significantly enhanced the expression levels of Orai1 and Orai2, but not of Orai3 (Figure 2A–D). Our functional assay results indicated that PSG9 treatment also markedly increased the SOCE evoked by TG-induced Ca^2+^ store depletion in HUVECs and that this increased SOCE was nearly completely abolished by pretreatment with the Orai inhibitor BTP2 (0.5 μM) (Figure 3). These findings indicated that PSG9 increased SOCE by stimulating increased expression levels of Orai1 and Orai2 in HUVECs.

### 3.3. Role of Orais in PSG9-Enhanced eNOS Expression and NO Production in HUVECs

**Figure 4:**
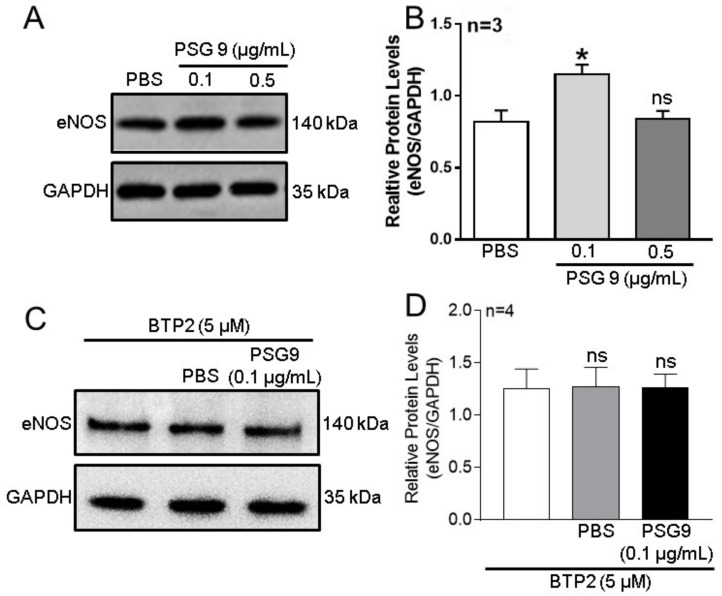
Effect of PSG9 on endothelial nitric oxide synthase (eNOS) expression level in human umbilical vein endothelial cells (HUVECs). (**A**–**D**) Representative Western blot images (**A**,**C**) and summary data (**B**,**D**) showing the expression of eNOS in HUVECs incubated with PBS or different concentrations (0.1 or 0.5 µg/mL) of PSG9 for 24 h (**A**,**B**), or with BTP2 (5 μM) + PBS or BTP2 (5 μM) + PSG9 (0.1 μg/mL) (**C**,**D**). Values are shown as the mean ± SEM. * *p* < 0.05 compared with PBS; ns, no significant difference.

Endothelial cells express eNOS to synthesize NO, an important NO source to regulate vessel tension [16]. The Ca^2+^ signal is crucially involved in the regulation of eNOS expression and NO production [16,20,21,22]. Preeclampsia is pregnancy-induced hypertension characterized by systemic microvascular spasm. Therefore, to investigate the possible effect of Orai-mediated SOCE on eNOS expression and NO production, we used Western blotting assays to assess the changes in expression levels of eNOS in HUVECs incubated with different concentrations of PSG9 for 24 h. The results showed that compared with the effect of PBS, the application of PSG9 at a concentration of 0.1 μg/mL significantly enhanced eNOS expression levels in HUVECs, but this enhancement was not observed when the PSG9 concentration was increased to 0.5 μg/mL (Figure 4A,B). In addition, when cells were pretreated with BTP2 (5 μM), the enhanced eNOS expression induced by the lower concentration of PSG9 (0.1 μg/mL) was completely abolished (Figure 4C,D).

**Figure 5:**
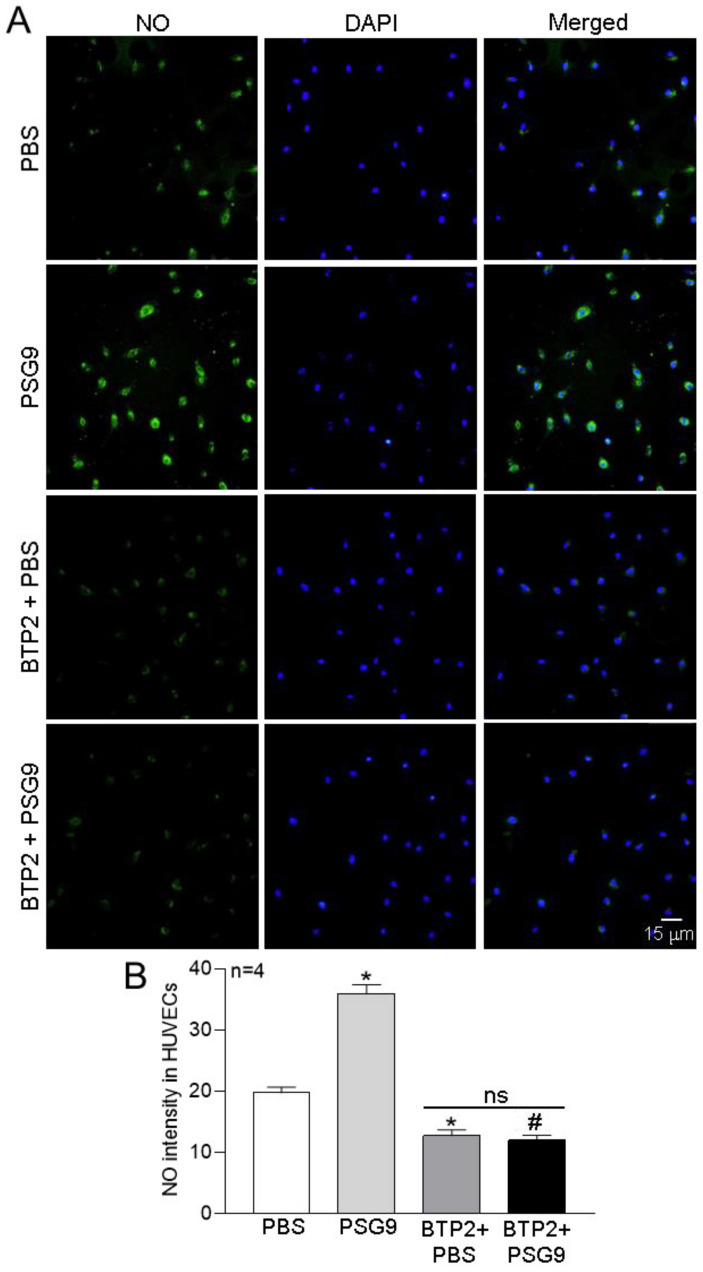
Effect of PSG9 on nitric oxide (NO) production in human umbilical vein endothelial cells (HUVECs). (**A**,**B**) Representative fluorescence images (**A**) and summary data (**B**) showing NO levels (green fluorescence) in HUVECs treated with PBS, BTP2 (5 μM) + PBS, 0.1 μg/mL PSG9, and 0.1 μg/mL PSG9 + BTP2 (5 μM). The nucleus is indicated by blue fluorescence (4′,6-diamidino-2-phenylindole; DAPI). Values are shown as the mean ± SEM of fluorescence arbitrary units (*n* = 4 in each group). * *p* < 0.05 compared with PBS, ^#^ *p* < 0.05 compared with PSG9; ns, no significant difference.

To further explore the effect of PSG9 on NO production in HUVECs, the cells were incubated with 0.1 μg/mL PSG9 for 24 h. Then, NO was detected using the cell-permeable fluorescent probe DAF-FM DA, for which the fluorescence intensity reflects the content of NO in cells: the stronger the fluorescence, the higher the NO concentration. As shown in Figure 5, compared with the PBS control group, the intensity of the green fluorescence representing NO in the cells treated with PSG9 was significantly enhanced, indicating that PSG9 promoted the production of NO in cells. Moreover, BTP2 treatment significantly abolished the PSG9-enhanced NO production (Figure 5). These results indicated that PSG9 increased NO production through an increased eNOS expression induced by SOCE-mediated Ca^2+^ signaling in HUVECs.

## 4. Discussion

PSG9 is a highly conserved secreted cytokine that is produced mainly by placental syncytiotrophoblasts and secreted into the blood. It plays important roles in immune regulation, thrombosis regulation, and angiogenesis during pregnancy [24,25,26]. The concentration of PSG9 in a pregnant woman’s serum increases with gestational age and reaches its peak at the third month of pregnancy [27,28]. There is evidence that adverse pregnancy outcomes, such as fetal growth restriction, premature delivery, and preeclampsia, are related to reduced blood levels of PSG1 in pregnant women [11,29,30], but there are few reports examining PSG9. The major findings in the present study were as follows: (1) compared with those in healthy pregnant women, serum levels of PSG9 in pregnant women with preeclampsia were significantly lower; (2) application of PSG9 at a concentration of 0.1 μg/mL to HUVECs increased the expression levels of Orai1 and Orai2, but not Orai3, thus promoting SOCE; (3) application of PSG9 at a concentration of 0.1 μg/mL to HUVECs increased eNOS expression levels, which enhanced the level of NO production; (4) BTP2, an inhibitor of Orais, blocked this PSG9-induced eNOS expression and NO production in HUVECs. These results suggest that PSG9 may regulate endothelial cell function through a Ca^2+^/eNOS/NO signaling pathway, leading to changes in vascular tension, and that PSG9 may be associated with the occurrence or development of preeclampsia.

During normal pregnancy, trophoblast invasion, placental angiogenesis, vessel remodeling, and placental immune responses occur. A number of different communication methods are established between the mother and her fetus, inducing placental trophoblasts that produce a variety of signaling molecules. These molecules act on endothelial cells, T cells, NK cells, and other target cells to generate corresponding physiological effects [24,25,26,31,32]. Preeclampsia is a placental disease, thus the expression levels of certain secretion factors and molecular markers in placental trophoblasts and endothelial cells can be detected in maternal blood samples to monitor placental dysfunction [33]. The use of iTRAQ protein spectroscopy offers high sensitivity and high resolution [34]. By using iTRAQ technology, we previously found that the expression levels of PSG9 in the serum of pregnant women with preeclampsia were significantly decreased compared with healthy pregnant women [23]. In the present study, we confirmed that the PSG9 expression levels in the peripheral blood of patients with preeclampsia were lower than those in healthy pregnant women, consistent with the results from our previous iTRAQ study. PSG9 is a member of the pregnancy-specific glycoproteins secreted by gestational trophoblasts and is closely related to the maintenance of normal pregnancy at the beginning of pregnancy. Therefore, we speculate that this decrease in PSG9 levels may be associated with the occurrence of preeclampsia. However, Kandel et al. recently reported that PSG9 is significantly increased in preeclampsia [35]. There are at least four plausible reasons for the discrepant results between their study and ours. The first reason may be because of race and ethnicity differences. The study by Kandel et al. included persons from Australia, Africa, and Europe, whereas our study included only persons who were Han Chinese. The second reason may be because of differences in experimental methods. Kandel et al. used ELISA techniques, whereas we used iTRAQ protein profile, ELISA, and Western blotting methods. In addition, there may be differences in the specificities of the antibodies used in the two studies. A third reason may be because of sample differences. We assessed serum samples, whereas Kandel et al. assessed plasma. A fourth reason may be because of the influence of comorbidities among the participants in the study by Kandel et al. We strictly excluded participants with diseases that could affect serum proteins, such as infections and diabetes.

NO is a byproduct of nitric oxide synthase, which generates L-citrulline by using L-arginine and oxygen as substrates and nicotinamide adenine dinucleotide phosphate as a cofactor to provide electrons. NO synthesis is regulated by a variety of factors, including its substrates and products, but is mainly controlled by the enzyme nitric oxide synthase. Endothelial NOS (eNOS) is synthesized primarily by endothelial cells and plays an important role in promoting NO synthesis and regulating vascular tension. In normal pregnancy, blood flow of the maternal placenta must be maintained at a high level to ensure the nutrition and oxygen needed for fetal development and growth. The blood supply to the placenta can be improved by reducing the resistance of peripheral blood vessels. However, in preeclampsia, the ability of the placenta to vasodilate is disrupted. Through clinical experiments, Barr et al. have shown that the severity of preeclampsia is closely related to the diastolic reactivity of the vascular endothelium [36]. In the present study, we found that PSG9 at a relatively low concentration promoted the expression of eNOS and NO release in HUVECs, which may be linked to the maintenance of endothelial function during pregnancy. Ca^2+^ is an important regulator of many biological reactions, including regulating the production and release of NO in vascular endothelial cells [37]. An increase in intracellular Ca^2+^ in endothelial cells is mainly due to Ca^2+^ entry through the Na^+^/Ca^2+^ exchanger and R-type Ca^2+^ channels as well as Ca^2+^ release from the ER and nucleus [38,39]. Normally, a transient intracellular Ca^2+^ increase is due to Ca^2+^ released from the ER, whereas a sustained intracellular Ca^2+^ increase is due to Ca^2+^ entry through voltage and G protein-coupled receptor-gated Ca^2+^ channels. SOCE is a key pathway for extracellular Ca^2+^ influx, and its regulation of [Ca^2+^]_i_ homeostasis includes Ca^2+^ release from stores in the ER as well as Orai channel-mediated external Ca^2+^ influx. The former has a short timeframe, and the NO generated is only at trace levels, insufficient to cause biological effects. By contrast, Orai channel–mediated external Ca^2+^ influx can continuously increase [Ca^2+^]_i_. In addition, calmodulin (CaM) combines with Ca^2+^ to form a Ca^2+^/CaM complex, which then combines with the CaM binding domain in eNOS to prevent Thr495 of eNOS from being phosphorylated by protein kinase C, thereby more effectively promoting the synthesis of NO in the vascular endothelium [40]. Our results showed that PSG9 at a concentration of 0.1 µg/mL significantly enhanced the expression levels of Orai1 and Orai2, SOCE-mediated Ca^2+^ influx, and NO synthesis and release in HUVECs. Pretreatment with the SOCE channel protein inhibitor BTP2 effectively abolished PSG9-induced eNOS expression and NO production in HUVECs as well as SOCE-mediated increases in [Ca^2+^]_i_. However, a higher concentration of PSG9 (0.5 µg/mL) did not increase the expression of eNOS, suggesting that there may be dose-dependent effects of PSG9. That is, too high a concentration of PSG9 may produce other effects that ultimately do not promote increased eNOS expression. Takahiro et al. showed that Ca^2+^-sensing receptors may be involved in the production of NO in HUVECs and may be inhibited by receptor antagonists [41]. Moreover, Sun et al. used an ethanol extract of *Salvia officinalis* to increase the formation of NO in HUVECs, but when they removed extracellular Ca^2+^, NO production was inhibited [42]. The findings of those studies also support our hypothesis that the PSG9-induced enhancement of NO release in HUVECs may be due to the promotion of SOCE-mediated Ca^2+^ signaling. Therefore, PSG9 may regulate endothelial cell function through a Ca^2+^/eNOS/NO signaling pathway, leading to changes in vascular tension, and may be associated with the occurrence or development of preeclampsia. Although increased NO release typically relaxes vascular smooth muscle and contributes to hypotension, not to vasoconstriction, we measured only dynamic [Ca^2+^]_i_ changes in HUVECs treated with PSG9. We did not measure resting [Ca^2+^]_i_. Thus, the effect of resting [Ca^2+^]_i_ on cell function remains unknown. Future studies should also assess resting [Ca^2+^]_i_. 

In summary, we found that serum PSG9 levels were significantly decreased in pregnant women with preeclampsia. Further evidence showed that PSG9 promoted NO release by increasing the expression of Orai proteins and activating the Ca^2+^/eNOS/NO signaling pathway in HUVECs. In addition to clarifying a molecular mechanism whereby PSG9 regulates the function of vascular endothelial cells, these findings suggest that PSG9 may warrant further study for the prevention or treatment of preeclampsia.

## 5. Conclusions

In conclusion, this study showed that the levels of PSG9 in the serum of patients with preeclampsia were significantly decreased, and further experimental studies indicated that the Ca^2+^/eNOS/NO signaling pathway was mediated by increased Orai1 and Orai2 expression levels to enhance NO release in HUVECs. These findings suggest that PSG9 may have potential value in the prevention or treatment of preeclampsia. However, preeclampsia is a pregnancy-specific disease involving multiple factors and myriad cell types. This study clarified only the role of PSG9 in vascular endothelial cells to suggest its role in the pathogenesis of preeclampsia. The functions of PSG9 in trophoblasts remain uncertain and are also an important direction for future research.

## Figures and Tables

**Table 1:** Clinical data for patients with preeclampsia and healthy control participants.

Feature	Control Mean (SD) (*n* = 4)	Preeclampsia Mean (SD) (*n* = 4)	*t* Value
Age (years)	26.00 ± 1.83	24.75 ± 1.71	0.951
Gestational weeks (days)	272.25 ± 13.25	268.75 ± 7.63	0.458
SP (mmHg)	120.00 ± 8.04	167.50 ± 14.52 *	−5.722
DP (mmHg)	74.5 ± 9.47	105.00 ± 11.28 *	−4.141

Note: SP, systolic pressure; DP, diastolic pressure. Values are shown as the mean ± SD. * *p* < 0.05 for preeclampsia vs. control with the two-tailed Mann–Whitney test.

## Data Availability

Data will be made available by the corresponding author under reasonable request.
